# The Touch and Zap Method for *In Vivo* Whole-Cell Patch Recording of Intrinsic and Visual Responses of Cortical Neurons and Glial Cells

**DOI:** 10.1371/journal.pone.0097310

**Published:** 2014-05-29

**Authors:** Adrien E. Schramm, Daniele Marinazzo, Thomas Gener, Lyle J. Graham

**Affiliations:** Neurophysiology & New Microscopies Laboratory, INSERM U603 - CNRS UMR 8154, Université Paris Descartes, Paris, France; Consejo Superior de Investigaciones Cientificas - Instituto Cajal, Spain

## Abstract

Whole-cell patch recording is an essential tool for quantitatively establishing the biophysics of brain function, particularly *in vivo*. This method is of particular interest for studying the functional roles of cortical glial cells in the intact brain, which cannot be assessed with extracellular recordings. Nevertheless, a reasonable success rate remains a challenge because of stability, recording duration and electrical quality constraints, particularly for voltage clamp, dynamic clamp or conductance measurements. To address this, we describe “Touch and Zap”, an alternative method for whole-cell patch clamp recordings, with the goal of being simpler, quicker and more gentle to brain tissue than previous approaches. Under current clamp mode with a continuous train of hyperpolarizing current pulses, seal formation is initiated immediately upon cell contact, thus the “Touch”. By maintaining the current injection, whole-cell access is spontaneously achieved within seconds from the cell-attached configuration by a self-limited membrane electroporation, or “Zap”, as seal resistance increases. We present examples of intrinsic and visual responses of neurons and putative glial cells obtained with the revised method from cat and rat cortices *in vivo*. Recording parameters and biophysical properties obtained with the Touch and Zap method compare favourably with those obtained with the traditional blind patch approach, demonstrating that the revised approach does not compromise the recorded cell. We find that the method is particularly well-suited for whole-cell patch recordings of cortical glial cells *in vivo*, targeting a wider population of this cell type than the standard method, with better access resistance. Overall, the gentler Touch and Zap method is promising for studying quantitative functional properties in the intact brain with minimal perturbation of the cell's intrinsic properties and local network. Because the Touch and Zap method is performed semi-automatically, this approach is more reproducible and less dependent on experimenter technique.

## Introduction

The patch-clamp electrophysiological technique was developed to record currents from single membrane channels [1] and later applied to record macroscopic currents and voltages in the so-called whole-cell configuration [2]. Advances have allowed the study of functional synaptic dynamics, gene transcription at the cellular level, and measurements coupled with histology [2]–[7], for a review see [8]. Patch clamp recordings in intact tissue was made possible by Blanton and colleagues [4], who introduced the blind patch technique *in vitro*; this work inspired the first *in vivo* patch clamp recordings of functional responses in the intact animal [9], [10]. The introduction of *in vivo* two-photon microscopy has allowed visual monitoring of whole-cell recordings *in vivo* [11]–[15], but since this approach is limited to upper layers of the cortex, the blind patch method remains an important technique *in vivo*. The refinement of this technique has recently reached a new level, with the automated *in vivo* protocol described by Kodandaramaiah et. al. [16]. For reviews on the methodological aspect of the technique see [17]–[19].

Whole-cell patch recording is difficult to master, requiring a sequence of delicate maneuvers by the experimentalist. The *in vivo* setting introduces additional constraints; most importantly, maintaining the healthy physiological state of the animal can severely limit the “windows of opportunity” for recordings, and reducing brain movements is always a problem. In light of increasingly sophisticated *in vivo* protocols, such as simultaneous imaging with two-photon microscopy and the awake behaving preparation, these factors motivate simplifying the technical aspects of whole-cell patch protocols (e.g. obtaining the rapid access to the cell's interior). The limitation of positive pressure is further motivated when the pipette solution contains a dye, e.g., fluorescent calcium indicator [20], [21]. In this case, dye ejected from the pipette during the approach to the neuron increases the extracellular background fluorescence, reducing the contrast and limiting the number of attempts at a given cortical location [15], [22].

A constant challenge is to improve the fundamental step of obtaining electrical access to the interior of the cell, in particular to improve recording stability and to achieve low access, or “series”, resistance (R_a_, the resistance between the amplifier input and the cell interior), a crucial parameter for protocols that perturb membrane voltage with current supplied by the amplifier. Another concern is how the recording method modifies tissue or cell physiology. Previous methods to improve whole-cell patch recordings, for example the “tightness” of the seal, include cleaning the cell with either enzymes [2], or by applying positive pressure from the recording or an adjacent pipette [2], [4], [6], [17], [23], [24]. A similar “washing” is also performed by outflow of the pipette solution due to positive pressure while positioning the pipette on the cell membrane during *in vitro* or *in vivo* recordings under visual control (for example the “shadow” patching technique [14], [15]). In general, the standard protocol is to apply some type of “wash” step, obtain a gigaohm-seal by suction, and then achieve whole-cell access by applying a ramp or short pulses of suction to the pipette to stress the membrane patch underneath the pipette tip until it breaks. These hydraulic and mechanical operations may be detrimental: Outflow of intracellular solution with a high potassium concentration may initiate or intensify processes that change the dynamical state of the neuronal circuit, such as spreading depression [25], [26], or modify blood vessel contractility [27]. Histological examination of cortical tissue after *in vivo* patch recordings often shows significant physical damage due to the patch pipette, which will be exacerbated by solution outflow. Subjecting the membrane to directed flow from the pipette may also alter membrane protein function, if only by physical disruption. Finally, the essentially mechanical step of rupturing the membrane to obtain whole-cell mode by suction is difficult, if not impossible, to control at the microscopic level, compromising reproducibility and risking harm to the recorded cell.

To address these issues for whole-cell patch recordings, thus to simplify the technique, improve recording quality, and be less invasive to the recorded cell and its local network, we have developed a revised protocol, “Touch and Zap”. As presented here this method is a direct modification of the standard blind whole-cell patch method for *in vivo* cortical recordings, and is applicable to either blind or visually-guided patch clamp protocols in brain tissue, *in vitro* or *in vivo*.

## Materials and Methods

### Ethics statement

Protocols were approved by the “Direction Départementale des Services Vétérinaires de Paris”. All painful manipulations (incisions, pressure points) were preceded by injections of local anesthetic (lidocaine).

### Animal preparation

Adult Sprague Dawley Rats (male, 300–500 g) were anesthetized with urethane (1.5 g/kg i.p.). Fully anesthetized (no reaction to paw pinching; corneal reflex absent) rats were placed in a stereotaxic rig (Narishige SN-3N). Dexamethasone, to prevent cerebral edema (1 mg/kg i.m.), and either glycopyrrolate (∼0.03 mg/kg i.m.) or atropine methyl nitrate (0.3 mg/kg, i.m.), to reduce secretions and to prevent bradycardia, were injected at the beginning of the experiment. Rectal temperature was monitored and maintained at 37.5±0.5°C by a controlled heating blanket (CWE TC 1000). The electrocardiogram (ECG) was monitored to indicate the depth of anesthesia and the overall health state of the animal, and supplemental urethane was injected i.p as necessary.

For the cat protocols, anesthesia was induced in young adult males (3.2–4.2 kg) with a ketamine-xylasine mixture (10 and 1 mg/Kg respectively, i.m.). After tracheal intubation and installation of a urinary catheter and a rectal temperature probe the animal was placed in the stereotaxic rig (Narishige SN-3N). Rectal temperature was monitored and maintained at 37.5±0.5°C by a controlled heating blanket (CWE TC 1000). Throughout the experiment the animal was perfused by a propofol-sufentanil solution in Ringer 5% glucose (respectively, 5 mg/kg/h for anesthesia and 4 µg/kg/h for analgesia). Perfusion was adjusted as necessary to maintain anesthesia depth as monitored by the ECG and the pCO2 of expired air. To eliminate eye movements, paralysis was induced by pancuronium (0.3 mg/kg i.v.) followed by a continuous venous perfusion of a Ringer 5% glucose-pancuronium solution (0.3 mg/kg/h), and the animal was ventilated by a respiratory pump. A bilateral pneumothorax was performed to reduce brain movements.

After installation in the stereotaxic rig, craniotomies were performed, either above primary somatosensory cortex (for the rat) or above primary visual cortex (for both animals). The typical duration of the experiments was 4–6 hours for rats and from one to three days for cats. At the end of an experiment euthanasia was accomplished with an overdose injection of sodium pentobarbital (i.p. or i.v.).

### Electrophysiology

Patch electrodes were pulled from thin wall (OD 1.5 mm, ID 1.10 mm) borosilicate glass capillaries with filament (Sutter Instruments), using a horizontal puller (Sutter Instruments P-97) in 3 steps in order to have a small tip (∼2 µm) and a long and thin taper to minimize damage to the cortex. Pipette shape was constrained to have a resistance between 4 and 8 MΩ, when filled with intracellular solution containing (in mM unless indicated): potassium gluconate 140, potassium chloride 4, 4-(2-hydroxyethyl)-1-piperazineethanesulfonic acid (Hepes) 10, magnesium chloride 2, adenosine-5′-triphosphate dipotassium (ATP) salt 4, guanosine-5′-triphosphate (GTP) lithium salt 0.4, ethylene glycol tetraacetic acid (EGTA) 0.5 and in some experiments, 0.01% dimethyl sulfoxide (DMSO) and 1,2-bis(o-aminophenoxy)ethane-N,N,N′,N′-tetraacetic acid (BAPTA) tetrapotassium salt 10 or 5. The osmolarity of the intracellular solution was balanced to 285–295 mOsm with distilled water and pH adjusted to 7.4 with concentrated potassium hydroxide.

The recording system was specifically designed to make dynamic-clamp and visual protocols, and is fully described elsewhere [28]. Briefly, current clamp recordings under bridge mode were made with a Dagan Instruments BVC-700 intracellular amplifier. The membrane voltage and current output were low-pass filtered at 10 kHz and acquired at 40 kHz. Experiment control, data acquisition and data analysis were accomplished with in-house software using the LabView programming environment (National Instruments). Visual stimuli were generated using the VisionEgg software library [29].

### Electrode cortical insertion

Whole-cell patch electrodes were introduced into the cortex using a motorized micromanipulator (Narishige SM-21), with the electrode impedance monitored continuously with a 50% duty cycle, 10 Hz current pulses alternating between 0 and −1.11 nA (this value was chosen to allow rapid decade reduction of current amplitude). The manipulator was typically adjusted so that the pipette entered the cortex at an angle between 20 and 30 degrees off the perpendicular. For the initial descent, contact with the cortical surface and penetration, the electrode was advanced in continuous mode (∼750 µm/second), with a strong positive pressure of 100–300 mmHg applied to the interior of the pipette. After penetration of the cortical surface was detected by a transient deflection of the measured voltage, the electrode advance was continued until a predetermined depth (100–2000 microns) was reached. The electrode was then immediately retracted 100–200 microns in continuous mode, and the positive pressure quickly reduced to 40–60 mmHg (the step pressure P_step_) for advancing stepwise into the tissue. The electrode resistance was then determined by visual inspection and fully compensated by the bridge circuit of the amplifier, and voltage offset adjusted according to an estimated tip offset potential (−14 mV with the K-gluconate based solution used here).

### Whole-cell access

**Figure 1:**
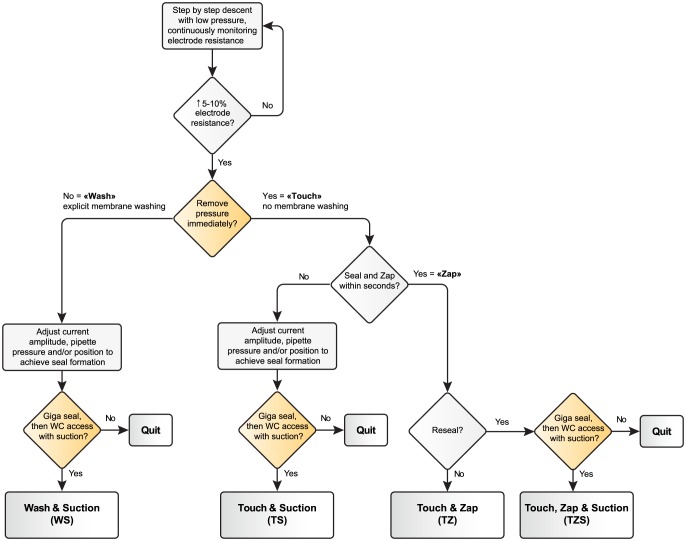
Summary flowchart for the whole-cell access methods described in the text. The orange diamonds highlight an explicit decision point by the experimenter (e.g., the first orange diamond depends on the choice to employ the WS method or a Touch and Zap variant), whereas the gray diamonds depend on the observed recording properties.

Subsequent manipulations to achieve whole-cell access mode (Figure 1) followed either the classical blind technique described in the literature (first described by Blanton and colleagues [4]), which we refer to “Wash and Suction” (WS), or with the Touch and Zap protocol. In either case the electrode was advanced in 3–4 micron steps through the cortex until a small deflection of the voltage response was observed (5–10% of the unbalanced response to the −1.11 nA pulses thus, given our electrodes, on the order of several hundred microvolts corresponding to a resistance increase of a few hundred kΩ), indicating contact with the cell membrane. If no change of the electrode resistance was detected after stepping several hundred microns, P_step_ was reduced by 5–10 mmHg, and the descent continued. This adjustment was repeated as necessary, unless the attempt was abandoned and the electrode replaced. Thus, the range of P_step_ values reported here represent an upper bound for a minimum value of this parameter, therefore even lower values, preferable to reduce the tissue perturbation, may be applicable for the Touch and Zap method (for example 18–26 mmHg used by Margrie and colleagues [17] for blind patching, or 5–15 mmHg for two-photon visually-guided patching in vivo (A. Schramm and P. Kara, and L.J. Graham, unpublished data).

A key subtlety at this step was to distinguish between an increase in electrode resistance due to contact with a cell, and that due to other causes. In particular, it was critical that the electrode tip be clear before contact with the target cell, assuming that the surface of the electrode tip must be uncontaminated to allow the molecular-level interaction between glass and lipid membrane that underlies the gigaohm-seal. For example, and in contrast with other reports (for example see [13], [17], [30]), in our experience a cardiac or respiratory artifact, possibly due to a blocked electrode or excessive tissue movement, either during the descent or in on-cell configuration (with gigaohm-seal or not), was associated with a poor intracellular recording. In general if we observed fluctuations of the electrode resistance on the order of 20%, the recording attempt was abandoned and a new electrode was used.

Once the electrode reached the target cell, the next step differentiated between the WS and Touch and Zap methods (Figure 1). For the standard WS method the positive pipette pressure was maintained on the order of ten seconds in order to “wash” the membrane. In our experience, a stable electrode resistance during this period (after the increase on contact), that can be easily modulated with small pressure changes (±10–20 mmHg), is correlated with successful seal formation and whole-cell access. After the “wash” the pressure was removed, and a giga-seal formed typically with the aid of light suction applied to the pipette. During seal formation the amplitude of the current steps was progressively reduced from −1.11 nA to −110 pA to −10 pA to avoid large voltage changes (e.g. >150 mV). When a stable gigaohm-seal was achieved, punctuate suction pulses were applied by mouth to achieve whole-cell access. If strong suction was required in order to obtain a gigaohm-seal, subsequent whole-cell access was more difficult and usually had a large access resistance.

The Touch and Zap method specifically avoids the “wash” step of the standard WS method. Thus, the pipette pressure was removed immediately on cell contact – the “Touch” – and under good conditions a seal began to form spontaneously. Also in distinction to the WS method, no (or very little) mouth suction was applied to the pipette *a priori* at this point. In fact, given the normal intracranial pressure of between 5 and 10 mmHg [31], [32], versus the pressure of the pipette interior, the released of the applied pipette pressure likely results in a small but significant negative pressure gradient across the pipette tip, thus an “automatic” suction.

In contrast to the WS approach, during seal formation the hyperpolarizing current pulses (initially used to monitor the electrode resistance) were maintained at −1.11 nA, which had two effects. First, because seal formation is facilitated by hyperpolarized membrane potentials [17], [33] a positive feedback was established, since voltage deflections became increasingly hyperpolarizing as the seal resistance increased. Second, given the magnitude of the resistance increase, the voltage responses to −1.11 nA could reach the breakdown voltage for the cell membrane within a few seconds, and whole-cell access was achieved by automatic electroporation – the “zap”. In about 25% of the recordings the access resistance seen by the electrode after the zap was close to the final value; in the remainder a smaller second zap followed within a few seconds (typically between at a potential between 100–150 mV less hyperpolarized than the first zap) which reduced the resistance further, again close to the final configuration. When whole-cell access was stable, we defined this endpoint as “pure Touch and Zap” (TZ, see Figure 1 and Results for examples). In some cells the seal quickly reformed after the first or second zap to a giga-seal configuration, and in that case the seal was re-broken with mouth suction. If stable access was achieved, we refer to this endpoint as “Touch, Zap and Suction” (TZS, see Figure 1 and Results; although not used here, we may note that in principle, re-establishing whole-cell access could be attempted by another zap). Finally, if a seal failed to form spontaneously within few seconds after the initial release of pressure (i.e. no zap) we then applied a constant hyper-polarizing current and/or pressure manipulations, as in the standard method. Then, if these procedures resulted in the establishment of a true giga-seal, whole-cell access was achieved by suction, similar to the WS method. If a stable recording was reached we referred to the endpoint as “Touch and Suction” (TS, see Figure 1 and Results). Interestingly, we found that if suction was needed for seal formation at any point, it was rare to see a successful zap.

The achieved endpoint out of the three possibilities following the touch step was in part automatic, and in part dependent on the experimentalist. TZ access was the most straightforward, being almost completely automatic, since no intervention has to be done by the experimentalist between the pressure released right after the touch and the whole cell access. Likewise, the TZS access was essentially imposed by the cell in question – either there was a resealing after the initial zap, or not. Using the TS approach for a given recording was more subjective, specifically deciding when to abandon spontaneous seal formation and resort to more classical manipulations. For our work, we typically chose TS access when there was only a slow increase in resistance, for example no electroporation many seconds after releasing the pipette pressure. Because this decision to follow the TS protocol was essentially arbitrary, the number of TZ and TZS recordings reported here is a lower bound on the number of cells which would have been electroporated if the zap had been tried for longer.

### Electrode compensation

Once stable whole-cell access was achieved, the amplifier capacitance compensation was adjusted to accelerate the fast electrode component as much as possible without oscillations during voltage responses to −100 to +300 pA subthreshold 10 Hz current steps. On-line R_a_ compensation using the amplifier bridge circuit was then adjusted using the same stimulus by the standard method of visually estimating the inflection point of the response that distinguishes the fast electrode artifact component from the (normally slower) passive membrane charging curve.

At this stage, application of a constant small positive pr*essure* (10–20 mmHg) to the pipette often reduced R_a_, although in our experience this tended to shorten the recording duration. R_a_ was typically estimated and compensated several times over the first minutes of the recording, with subsequent estimates made at intervals of several minutes.

### Electrophysiological and recording parameters, and data analysis

For off-line analysis we obtained the linear cell parameters with a heuristic inspired by the standard on-line visual method for bridge correction mentioned above, including resting potential V_rest_, the cell membrane time constant τ_0_, the uncompensated R_a_ and the cell input resistance R_in_. An often unappreciated aspect of the visual approach is that the fast time constant of the electrode (and bridge compensation circuit) is implicitly ignored, in contrast with direct methods that model the electrode, such as fitting a two exponential expression to the entire current step response [34], [35]. The intuition is not altogether misplaced: Not only does the short duration of the artifact compromise fit confidence, in any event it is the electrode resistance, not time constant, which is essential for balancing the bridge.

We considered 4 to 10 current step responses (0.5 second hyperpolarizing or depolarizing current) for each cell, where the steady-state response was limited to about ±10 mV from rest, without strong synaptic activity nor action potentials or other obvious non-linearities, for example sags. We assumed that the fastest time constant of the response was due to the electrode, and that the upper bounds of this and the cell time constant were a few hundreds of microseconds and tens of milliseconds, respectively. Assuming that the electrode time constant was one or two orders of magnitude smaller than the cell allowed the cell response fit to discount the electrode artifact by simply ignoring the first part of the response. Assuming an upper bound on the cell time constant allowed an independent estimation of the steady-state voltage, and permitted the estimation of cell time constant to focus on the actual transient. Finally, we assumed the step response could be reasonably approximated by a single exponential. This last assumption was made for simplicity, and improving the method with higher order fits will be considered in future work.

V_rest_ was first estimated by averaging periods of the membrane voltage prior to the application of stimulus current, rejecting periods that showed spontaneous spiking activity. The steady-state response of each trace, V_ss_, was then estimated as the average voltage over 100 milliseconds, starting 100 milliseconds after stimulus onset. The voltage just prior to the current step, V_O_, was estimated by a third order polynomial fit (to account for rapid membrane fluctuations) to the voltage over the two milliseconds just before stimulus onset. The trace voltage V was shifted to obtain V′ = (V_ss_−V) for depolarizing stimuli or V′ = (V−V_ss_) for hyperpolarizing stimuli. A first estimate of τ_0_ was then obtained by linear regression of the logarithm of V′ from 0.5 milliseconds after stimulus onset (thus after much of the electrode artifact had decayed), until V′ reached 0.3(V_ss_−V_rest_) for depolarizing stimuli or 0.3(V_rest_−V_ss_) for hyperpolarizing stimuli. A second iteration was made to refine the estimate by better isolation of the cell component, with the start of the V′ fit now set to stimulus onset plus 20% of the first estimate of τ_0_. The response fit was then extrapolated to stimulus onset and compared to V_O_. This voltage difference, divided by the stimulus amplitude, gave the on-line R_a_ compensation error (about 4 MΩ on average for all cells in this study), and the trace voltage was adjusted as necessary. R_in_ was obtained for each trace by the difference between V_rest_ and V_ss_ adjusted by the R_a_ error, divided by the amplitude of the current step. The linear parameters of the traces were then averaged to obtain the values for the cell. Mean R_a_ for a recording, and the R_a_ slope, ΔR_a_/Δt, corresponding to the evolution of R_a_ during the recording, and thus stability, were obtained by a linear regression of R_a_ estimates at various time points of the recording.

For all cells an initial IV protocol was made to establish basic intrinsic biophysical properties, including input impedance and membrane time constant as described above, as well as non-linear responses to establish cell type. The latter classification was based entirely on voltage responses to 500 millisecond current steps; cells with classical current-evoked action potentials of at least 50 mV in amplitude (measured from the resting potential) were classified as neurons, specifically into four physiological types as described by Nowak et al (2003) [42], thus, regular-spiking, fast-spiking, intrinsic bursting and chattering cells. Cells with no obvious fast voltage-dependent properties (time constant <5 ms) were classified as glial cells. The remaining recordings were not considered further in this study. This physiologically-based criteria, thus spiking or not, is standard for distinguishing glial cells and neurons, but since the detailed in vivo electrophysiology of glial cells is less well known we consider this classification as putative. A subset of the neurons and glial cells reported here were tested for visual responses, typically with stimulus sets composed of full field or circumscribed moving sinusoidal gratings (described in [28]).

Recording durations were determined by extrinsic and intrinsic factors. The former was defined by the specific experimental protocol, the shortest being a simple measurement of the voltage response to imposed current steps as described above (minimum recording duration of about one minute), and the longest aimed at measuring and manipulating visual responses. For glial cells, only a subset of cells were tested for visual properties, and of those typically only one to five visual protocols were made, each with a duration of about one minute. Intrinsic factors included the stability of the cellular and electrode properties. First, if there was a large R_a_ (typically greater than 50 MΩ) following whole-cell access, then the recording was abandoned after a single IV protocol. For stable recordings, a recording was usually terminated if the value of R_a_ increased more than 50% of its initial value (the most likely cause), or if the resting potential strayed more than ∼20 mV from its initial value, or if there were large (many millivolt) movement artifacts in the voltage trace. If R_a_ was not verified systematically during the longer visual response protocols, recording duration took into account stable visual responses, including spike heights for neurons within 20 mV of that measured at the beginning of the recording.

The recording depth was taken as the distance of the electrode trajectory between the cortical surface (indicated by a sharp deflection of the electrode voltage during the initial, fast descent) and the recorded cell. This represents an upper bound of the true trajectory length because of dimpling of the cortical surface due to the electrode insertion. The actual cortical depth of the recordings was not measured systematically, but can be estimated taking into account the angle of the electrode relative to the cortical surface, as described above.

For the TZ and TZS protocols, we measured the delay or “zap delay” (T_zap_) between the release of the pipette pressure and the abrupt reduction of the resistance seen by the electrode. We also report the peak hyperpolarization or “zap voltage” (V_zap_) achieved by the −1.11 nA pulses.

When comparing between whole-cell access methods, unless otherwise indicated, significant differences in the mean among pair-wise data sets were assessed using MANOVA. Statistical significance in the difference between parameter means taken over all neurons and all glial cells was established by a two-tail Student T test, and significant differences between proportions of neurons versus glial cells for the WS and the Touch and Zap methods was evaluated using the two proportion z test. Unless indicated, results are presented as sample average ± the standard deviation, with the sample population in parentheses.

## Results

Results were obtained from 372 whole-cell patch-clamp recordings (out of approximately 1800 attempts, with one attempt per electrode). Quantitative analysis was made on a subset of these cells for which at least one current clamp protocol (for example firing rate versus input current curve) have been performed. Two glial cells with very high input resistances (>500 GΩ) were excluded from further analysis. For the remaining cells (N = 201), 33 were recorded with the WS method, and 168 with the Touch and Zap method including 87 recordings with the TZ endpoint, 60 with the TZS endpoint and 21 with the TS endpoint (separated by species and cell type in Table 1). While the WS method has been used extensively by the corresponding author [36], [37], the WS cohort presented here was obtained at an early stage of this study for comparative purposes, and only from rat recordings; the Touch and Zap method was developed over the course of this study on both rat and cat. In general, there were no significant differences in measured properties as function of species; unless mentioned neuron and glial cell recordings are pooled across species.

**Table 1:** Total number of recorded neurons and glial cells for the rat and the cat, as a function of whole-cell access method.

		WS	TZ	TZS	TS	TOTALS
Rat	Neuron	28	22	14	3	67
	Glia	5	8	3	2	18
Cat	Neuron	n/a	37	35	11	83
	Glia		20	8	5	33
	TOTALS	33	87	60	21	201

“Wash and Suction” (WS), “Touch and Zap” (TZ), “Touch, Zap and Suction” (TZS), and “Touch and Suction” (TS). In the subsequent tables, unless noted, results from rat and cat neurons, and from rat and cat glial cells, respectively, are pooled.

### Relative Frequency of Touch Variants

For the protocols that commenced with the touch step, the first observable concerns the frequency among the possible TZ, TZS and TS endpoints. Interestingly, the ranking of the variants was the same for neurons and glial cells. TZ whole-cell access was most frequent, occurring in 48% of the neuron and 61% of glial cell recordings. The TZS variant was the next most frequent, with 40% of neuron and 24% of glial cell recordings, and TS recordings were obtained in 12% of the neurons and 15% of the glial cells. From this result, it can be seen that fast and spontaneous seal formation, with whole-cell access initiated by electroporation (i.e. TZ and TZS) was observed in most cells subsequent to the touch step, thus 89% of the neuron and 73% of the glial cells. Because the proportion of TS recordings was small and, as explained above, depended on experimenter judgment, unless mentioned the following results consider only the TZ and TZS endpoints of the Touch and Zap method. Since the relative numbers of recordings of neurons versus glial cells with the TZ and TZS endpoints were similar between rat and cat (Table 1), this supported grouping results for the two species unless otherwise mentioned.

The second observation regards how the recording approaches influence the proportion of neuron versus glial cells in blind patch protocols. Our results suggest that protocols starting with the touch step increase the chances of recording glial cells: the proportion of glial cell recordings following the touch step (46 out of 168 recordings, or 27%), was significantly greater than recordings using the WS method (5 out of 28 recordings, or 15%).

### Relative Proportions of Neuron Physiological Types and Glial Cells

In Table 2 we report the numbers and relative proportions of physiological neuron types and glia cells recorded over all the Touch and Zap variants (thus, TZ, TZS and TS) in the rat and in the cat. For comparison, this table includes the relative percentages of corresponding physiological neuron types as reported by Nowak et al (2003) [42], from adult cat visual cortex in vivo using sharp microelectrodes. Of note is that the relative populations of cat neurons in this study are consistent with the study by Nowak et al (2003) [42] (no statistical difference using Z test for 2 population proportions). In addition, we did not identify chattering cells in our rat recordings, a negative finding which has been noted by previous workers (ref. Nowak et al, 2003 [42]). Indeed, the fact that the cat and rat protocols reported here were essentially identical and contemporaneous argues against the existence of chattering cells in rodent cortex.

**Table 2:** Relative proportion of neuron physiological types and glial cells in the rat and cat for the WS and pooled Touch and Zap methods (TZ, TZS and TS).

		Rat (TZ+TZS+TS)		Cat (TZ+TZS+TS)		Cat (Sharp, Nowak et al, 2003)	
Cell Type		Total	% of Neurons	Total	% of Neurons	Total	% of Neurons
Neurons	FS	3	7	16	19	33	15
	RS	34	81	29	34	91	41
	CH	0	0	16	19	31	14
	IB	5	12	24	28	65	30
		Total	% of All Cells	Total	% of All Cells		
Glia		13	24	33	28		

For comparison this table includes the relative proportions of neuron physiological types as reported by Nowak et al (2003) [42] in the cat using sharp microelectrodes in vivo. There is no significant difference between the relative proportions of neuron types in the present study and that reported by Nowak et al (2003) [42] (p>0.5). Abbreviations: FS, fast-spiking; RS, regular-spiking; CH, chattering; IB, intrinsic bursting.

### Example Recordings with the Touch and Zap Method

**Figure 2:**
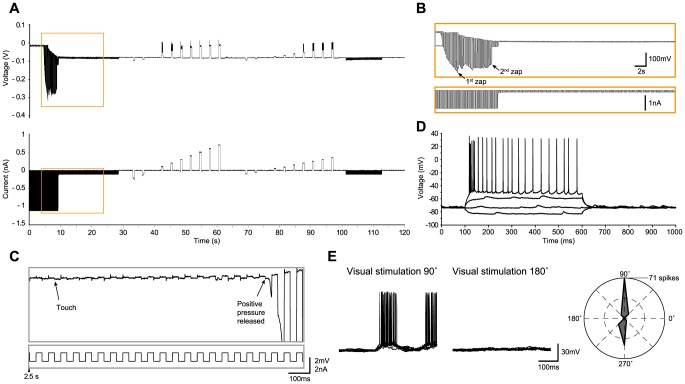
Regular-spiking neuron in cat visual cortex *in vivo* recorded with the TZ method. (A) Voltage and current recordings under current clamp mode during the approach to the neuron, detection of contact (“touch”; at ∼6 seconds), release of pressure with immediate seal formation, electroporation of the membrane and establishment of whole-cell access (“zap”), and initial measures of the voltage response to current steps. (B–C), expanded traces from (A) highlighting the access to the whole-cell configuration. (B) corresponds to the region marked by the orange square in (A), and (C) corresponds to the region marked by the grey square in (B). The “touch” is characterized by a small increase in the electrode resistance (C). The positive pressure in the pipette is released within a second after the touch, followed by an immediate increase in the electrode resistance as the seal spontaneously forms. A first “zap” occurs within about one second (B), followed by a second “zap” which results in the whole-cell configuration, evidenced by the much smaller voltage deflections, and the hyperpolarization of the voltage envelope as the cell's resting potential establishes a DC bias. At this point the current pulses are typically set to 100 pA, switching polarity as necessary to estimate and compensate for R_a_. (D) Voltage responses to current steps (−100, 0, 150 and 250 pA), with the response to 250 pA showing a regular-spiking firing pattern. (E) Left – Examples of preferred (180°) and non-preferred (90°) responses (10 trials overlaid) to a moving sinusoidal grating (4 Hz, 100% contrast, 0.5 second duration). Right - Total spike count over the 10 trials as a function of stimulus direction, showing that this neuron is strongly tuned to stimulus orientation and modestly tuned to stimulus direction. The on-line corrected R_a_ for this recording was 24 MΩ, which was subsequently corrected off-line to 21 MΩ. The input resistance was 102 MΩ, giving a relative R_a_ of 0.21.

**Figure 3:**
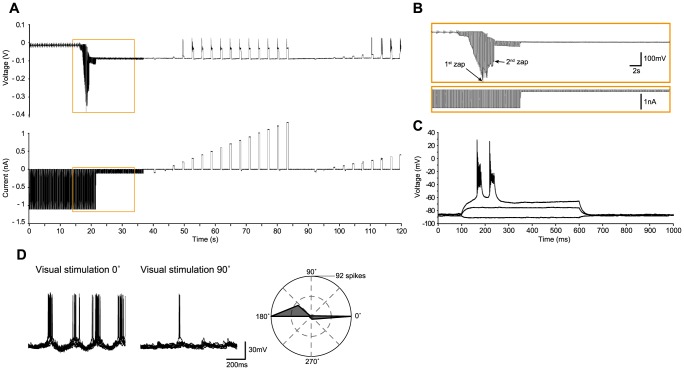
Bursting neuron in cat visual cortex *in vivo* recorded with the TZ method. (A–B). As in Figure 2, voltage and current recordings during the approach to the neuron, the “touch” (at ∼16 seconds), release of pressure with immediate seal formation, the first “zap” about two seconds later, followed by a second “zap” and establishment of whole-cell access, and initial measures of the voltage response to current steps. (C) Voltage responses to current steps (−50, 150 and 250 pA), showing a bursting firing pattern in response to the 250 pA step. (D) Left – Examples of preferred (0°) and non-preferred (90°) responses (10 trials overlaid) to a moving sinusoidal grating (4 Hz, 100% contrast, 1 second duration). Right – Total spike count over the 10 trials as a function of stimulus direction, showing the strong orientation tuning of this neuron. The on-line corrected R_a_ was 44 MΩ, which was subsequently corrected off-line to 61 MΩ. The input resistance was 70 MΩ, giving a relative R_a_ of 0.87.

**Figure 4:**
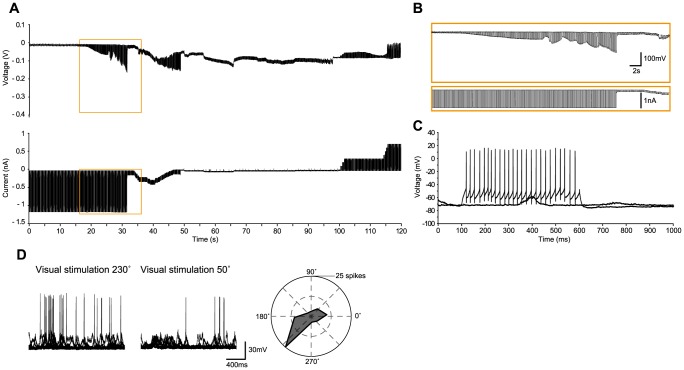
Fast-spiking neuron in cat visual cortex *in vivo* recorded with the TS method. (A–B). As in Figure 2, voltage and current recordings during the approach to the neuron, with the “touch” at ∼36 seconds. As can be appreciated in (B), and in distinction to the TZ endpoints, here there is only a slow increase in electrode resistance after the release of the pipette pressure. At approximately 51 seconds, the “zap” attempt is abandoned, and the standard methods of suction and hyperpolarization for obtaining a gigaseal were used, with the whole-cell access achieved by mouth suction at ∼70 seconds. (C) Voltage responses to current steps (0 and 800 pA), showing a fast-spiking firing pattern to the suprathreshold stimulus. (D) Left – Examples of preferred (230°) and non-preferred (50°) responses (10 trials overlaid) to a moving sinusoidal grating (4 Hz, 100% contrast, 1 second duration). Right – Total spike count over the 10 trials as a function of stimulus direction, showing that this neuron is mainly tuned to stimulus direction. The on-line corrected R_a_ was 37 MΩ, which was validated off-line. The input resistance was 26 MΩ, giving a relative R_a_ of 1.4.

**Figure 5:**
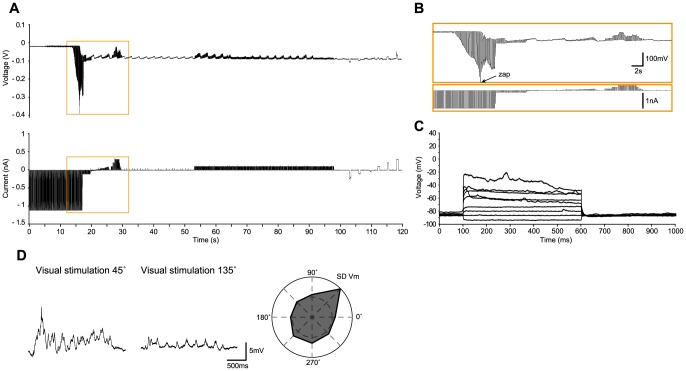
Glial cell in cat visual cortex *in vivo* recorded with the TZ method. (A–B). As in Figure 2, voltage and current recordings during the approach to the cell, the “touch” (at ∼33 seconds), release of pressure with immediate seal formation, the first “zap” about two seconds later. The current was reduced to 100 pA about one second later, and final whole-cell access was achieved by a small suction. As for neurons (e.g. Figures 2 and 3), the “zap” is distinguished by the sudden reduction in resistance while the strong hyperpolarizing current pulses are applied. (C) Voltage responses to current steps (100 nA increments from −100 to 700 nA), showing the lack of spikes and a fast membrane time constant that are characteristic of glial cells. (D) Left - Preferred (45°) and non-preferred (135°) current clamp responses (average of 10 trials) to a moving sinusoidal grating (4 Hz, 100% contrast, 2 second duration). Right – Average standard deviation of the visual responses (over the 10 trials) as a function of stimulus direction, showing that the membrane voltage fluctuations of this cell are tuned to stimulus direction. The R_a_ estimated on-line was 45 MΩ (validated off-line), and the cell input resistance was 105 MΩ, thus a relative R_a_ of 0.43.

**Figure 6 pone-0097310-g006:**
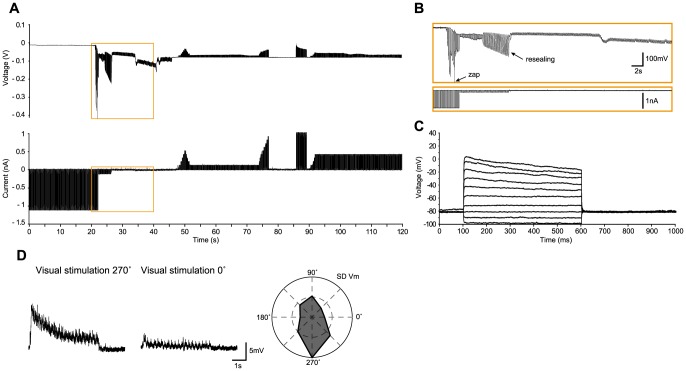
Glial cell in cat visual cortex *in vivo* recorded with the TZS method. (A–B) As in Figure 2, voltage and current recordings during the approach to the cell and subsequent whole-cell access. Here, an initial spontaneous seal with a subsequent “zap” was accomplished, but in distinction to the TZ endpoints illustrated in Figures 2, 3 and 5, the membrane resealed (between 24 and 27 seconds), and whole-cell access was re-established by suction at ∼46 seconds (not visible in (B)). (C) Voltage responses to current steps (100 nA increments from −200 to 700 nA). (D) Left – Preferred (270°) and non-preferred (0°) voltage responses (average of 10 trials) to a moving sinusoidal grating (4 Hz, 100% contrast, 5 second duration). Right – Average standard deviation of the visual responses (over the 10 trials) as a function of stimulus direction, showing that this cell is tuned to stimulus direction, with a small bias for stimulus orientation. The on-line corrected R_a_ was 40 MΩ, which was validated off-line. The input resistance was 99 MΩ, giving a relative R_a_ of 0.40.

We now present several examples of *in vivo* recordings with the different methods in the cat, including regular-spiking (Figure 2), bursting (Figure 3) and fast-spiking neurons (Figure 4), and three glial cells (Figures 5–7), illustrating the whole-cell access variants (Figures 2–6), basic measurement of intrinsic properties based on voltage responses to current steps, and visual responses. The examples, which show the TZ, TZS and TS endpoints, were chosen to highlight the diversity of recordings using the Touch and Zap method. To facilitate comparison between the endpoints, the beginning of each recording is plotted on the same scale (panels A and B of Figures 2–6). The voltage and current logs illustrated at the top of these figures were generally started when the stepwise descent into the cortex began, which gives an idea of the fairly short delay, usually less than one minute, between electrode insertion and obtaining a recording. In particular, the characteristic voltage envelopes for the different Touch and Zap endpoints can be appreciated. The envelope for the TZ endpoint (panel B in Figures 2, 3 and 5) is essentially monophasic, quickly growing as the seal forms, and then shrinking in one or two abrupt steps as electroporation achieves whole-cell access. The voltage envelope for the TZS endpoint (panel B in Figure 6) adds a second phase, with a slower increase as the cell re-seals on its own, followed again by an abrupt reduction as whole-cell access is re-achieved by mouth suction. Finally, the voltage envelope for the TS endpoint (panel B in Figure 4) develops much more slowly than the other two, finished by an abrupt reduction as whole-cell access is achieved by suction.

**Figure 7:**
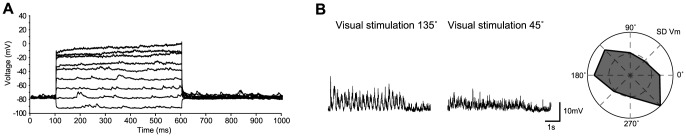
Gial cell in cat visual cortex *in vivo* recorded with the WS method. (A) Voltage responses to current steps (100 nA increments from −100 to 700 nA). (B) Left – Preferred (135°) and non-preferred (45°) voltage responses (average of 10 trials) to a moving sinusoidal grating (4 Hz, 100% contrast, 5 second duration). Right – Average standard deviation of the visual responses (over the 10 trials) as a function of stimulus direction, showing that this cell is tuned to stimulus orientation, with a small bias for stimulus direction. The on-line corrected R_a_ was 48 MΩ, which was validated off-line. The input resistance was 100 MΩ, giving a relative R_a_ of 0.48.

To illustrate the high quality recordings obtained with the Touch and Zap method each example also shows the intrinsic response to injected current steps (panel D in Figure 2 and panel C in Figure 3–6) and functional visual responses (panel E in Figure 2 and panel D in Figure 3–6), specifically the canonical selectivity of cells in primary visual cortex to the orientation or direction of a visual feature. We emphasize that the Touch and Zap method is not necessary to obtain whole-cell recordings from glial cells in vivo; Figure 7 shows the intrinsic (panel A) and visual responses (panel B) from a glial cell recorded with the WS method (but see the specific advantages of Touch and Zap below). Overall of particular note is the relatively fast dynamics of the glial visual responses in these examples, showing the ability of these cells to follow stimuli up to at least 4 Hz.

### Recording duration

**Figure 8:**
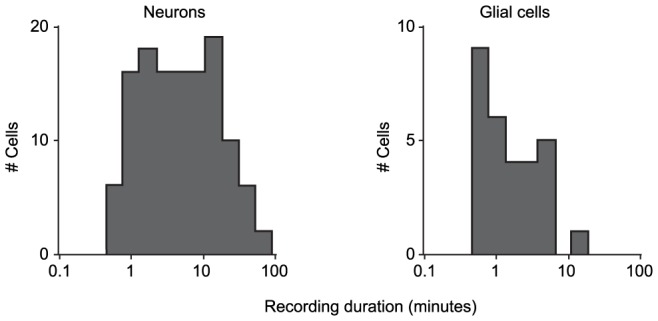
Distribution of recording durations for all neuronal and glial recordings in this study.

For the cells analyzed here, the mean recording duration was 12±17 minutes (N = 150) for all neurons, and 2.7±2.9 minutes (N = 51) for all glial cells, with a maximum duration of approximately 80 minutes and 15 minutes for neurons and glia, respectively (Figure 8).

### Recording and cell parameters

To further characterize the Touch and Zap method we now compare recording protocol parameters across methods, such as the electrode resistance, the pressure applied to the pipette during cell approach, the recording depth, the delay and minimum voltage of the zap step (Table 3). Then, to assess the impact of the method on cell physiology, we next compare cellular properties obtained using the Touch and Zap (pooled TZ and TZS populations) and WS methods, including the cell input resistance and membrane time constant, an estimate of the cell size based on input resistance, the resting potential, characteristics of action potentials for neurons, the absolute and relative access resistance, and the stability of the access resistance (Table 4).

**Table 3:** Protocol parameters for whole-cell patch recordings of neurons and glial cells *in-vivo*.

		Neuron	Glia
TZ+TZS+TS	R_e_ (MΩ)	6.0**±0.73 (121)	6.4**±0.58 (39)
	P_step_ (mmHg)	51±6.9 (115)	49±6.3 (36)
	Depth (µm)	874±345 (89)	863±423 (13)
TZ+TZS	V_zap_ (mV)	−330*±53 (91)	−350*±64 (34)
	T_zap_ (sec)	2.6**±1.9 (87)	4.2**±3.2 (33)

Electrode resistance (R_e_), step pressure (P_step_), recording depth, are reported for the pooled TZ, TZS and TS endpoints, and “zap” voltage (V_zap_) and “zap” delay (T_zap_) are reported for the pooled TZ and TZS recordings. Statistical significance in the difference of the means (entries in bold) is indicated by * (p<0.1) or ** (p<0.05), for this table (between cell types) and the following table (between recording method for a given cell type).

**Table 4:** Cell and recording parameters for cortical neurons and glial cells *in vivo* according to the WS and the pooled TZ and TZS whole-cell access methods.

	Neuron		Glia	
	WS	TZ+TZS	WS	TZ+TZS
τ_0_ (msec)	12±8.2 (28)	9.8±5.2 (103)	4.6±2.8 (4)	1.5±2.9 (35)
R_in_ (MΩ)	66±58 (28)	56±31 (108)	44±49 (5)	90±62 (39)
L_rel_	0.99±1.0 (28)	1.0±1.0 (108)	1.6**±1.4 (5)	0.92**±0.9 (39)
V_rest_ (mV)	−69**±9.5 (28)	−76**±7.5 (105)	−79±19 (5)	−76±13 (39)
dV/dt (mV/msec)	170*±69 (17)	230*±85 (28)	n/a	
V_peak_(mV)	7.4±9.1 (17)	11±9.3 (28)		
R_a_ (MΩ)	34±13 (28)	35±18 (108)	57±42 (5)	53±26 (39)
R_a_/R_in_	0.83±0.68 (28)	0.77±0.53 (108)	3.2±2.5 (5)	1.3±2.3 (39)
ΔR_a_/Δt (MΩ/min)	0.0±3.3 (24)	1.3±6.2 (101)	1.3±2.1 (5)	1.7±7.3 (24)

Parameters include the cell membrane time constant (*τ_0_*) and input resistance (R_in_), an estimate of the relative cell size L_rel_, the resting potential (V_rest_), the maximum depolarization rate (dV/dt) and peak (V_peak_) of action potentials of regular-spiking neurons in the rat, the absolute (R_a_) and relative (R_a_/R_in_) access resistance, and the stability of the access resistance (Δr_a_/Δt). Statistical significance in this table is indicated only when found for the ANOVA between the WS, and pooled TZ and TZS populations (this choice was justified because of the consistency of results between the TZ and TZS endpoints).

#### Electrode resistance

On average, the electrode resistance, R_e_, was significantly larger for glial cells than for neuron recordings, but in practical terms the difference of 0.4 MΩ was small (Table 3). Across whole-cell access methods, comparing the three pooled Touch and Zap endpoints with the WS approach, there was no significant difference in R_e_ for either glial cells or neurons.

#### Step pressure and recording depth

For the ensemble of Touch and Zap recordings, there was no significant difference in the average P_step_ between glial cells and neurons. (Table 3). The broad distribution of P_step_ for these recordings implies that successful whole-cell access did not depend critically on this parameter. P_step_ was not systematically recorded for the WS protocols, which precluded quantitative comparison with the Touch and Zap method, but the same range (40–60 mmHg) was used.

#### Zap delay and voltage

The average zap delay of the pooled TZ and TZS recordings, T_zap_, was significantly longer for glial cells than neurons (Table 3). This parameter was susceptible to the experimenter judgment: while standard practice was to abandon the zap attempt after a few seconds, occasionally the large current pulse was maintained for much longer. The distributions of T_zap_ for both cell types were therefore skewed, and thus the medians (3.2 and 2.1 seconds for glial cells and neurons, respectively) are more indicative of what to expect in practice. These values reflect the fast seal formation that is characteristic of this method, as compared to the standard WS protocol where the seal is typically formed over tens of seconds.

The average zap voltage for the pooled TZ and TZS recordings, V_zap_, was significantly lower for glial cells compared to neurons, with the overall values for both cell types consistent with reported values of the lipid bi-layer membrane breakdown voltage [39], [40] (Table 3).

#### R_in_ and *τ_0_*

There was no significant difference in either R_in_ or *τ_0_* for neurons or glial cells as a function of access method (pooled TZ and TZS endpoints, compared with WS; Table 4). This result provides partial validation of the gigaohm seal of the TZ recordings, which did not allow direct observation of the seal since whole-cell access is achieved when the seal is on the order of 300–400 MΩ. Nevertheless, the fact that R_in_ and *τ_0_* were independent of the access method supports the hypothesis that a conventional gigaohm-seal was fully formed after the zap, in lieu of a poor seal which would shunt or shorten the intrinsic R_in_ and *τ_0_*, respectively.

#### Estimation of relative Cell Size

Although the average R_in_ of glial cells did not depend significantly on the recording method, the average value and range of R_in_ from the pooled glial cell TZ and TZS recordings (90 MΩ, and from 3.2 to 280 MΩ, respectively) were more than twice those measures made with the WS method (44 MΩ, and from 6.7 to 110 MΩ, respectively). We tested the hypothesis that these differences could be explained by different target populations in terms of size, by comparing a first-order estimate of the relative (linear) sizes of the recorded cells from each method, L_rel_, defined as the square root of the reciprocal of R_in_, relative to the overall average for the cell type (Table 4). For glial cell recordings both TZ and TZS endpoints gave significantly smaller estimates of cell size than the WS method, with the average value of L_rel_ for the TZ and TZS glial cell recordings relative to L_rel_ for the WS glial cell recordings being 0.58. In addition, the span of L_rel_ for the TZ and TZS endpoints (0.40 to 3.7) encompassed that of the WS population (0.6 to 2.6). To clarify the importance of the initial touch step versus the zap step via-a-vis targeting a broader glial cells population, we repeated the analysis of mean L_rel_ with the TS population, finding it was also significantly smaller than the WS glial cells (data not shown). These results suggest that using the touch step was sufficient to increase the range of recorded cells. In contrast to the glial cells results, there was no significant difference in L_rel_ for neurons as a function of whole-cell access method.

#### V_rest_

The average resting potential of all neurons obtained with the WS method was significantly more depolarized than the pooled TZ and TZS endpoints. To control against species differences, we made the same comparison restricted to first rat and then cat neurons. Because the present data set did not include cat WS recordings, we referenced our published cat data for V_rest_ that used the WS method (−73±9 mV, N = 109, [36] and −67±6 mV, N = 49, [37]). We found that for both rat neurons and cat neurons, V_rest_ was significantly more hyper-polarized for the pooled TZ and TZS endpoints compared to the WS recordings (cat data tested with ANOVA, p<0.0005; Table 4). This significant difference may be explained by the longer perfusion of high potassium solution from the pipette for the WS as compared to the Touch and Zap method, since an increase in extracellular potassium would be expected to depolarize adjacent cells. Conversely, the average V_rest_ of glial cells recorded with the WS method (rat and cat data pooled), was more hyperpolarized than for the TZ and TZS endpoints, but this difference was not statistically significant.

#### Spike depolarization and peak of regular-spiking neurons in the rat

A relatively unadulterated signature of the overall functional health of the membrane channels may be the maximum depolarization rate and peak of the action potential, both of which being primarily determined by sodium channel dynamics and the passive properties of the membrane. We compared these parameters for spikes evoked by depolarizing current steps, restricting the analysis to regular-spiking neurons (as described in [41], [42]) in the rat to avoid confounding the results due to the various action potential characteristics of different physiological neuron types, and any systematic differences between species. On average, the maximum spike upstroke of all regular-spiking rat neurons was significantly faster between the pooled TZ and TZS endpoints as compared to the WS recordings. Consistent with this result, the spike peak of these neurons for the pooled TZ and TZS endpoints were on average higher than the WS recordings, although the differences were not statistically significant.

#### Access resistance and stability

We then compared the estimated access resistance, R_a_, both absolute and normalized to the cell's input resistance (thus R_a_/R_in_), and the change in R_a_ over time, ΔR_a_/Δt. Considering only glial cell recordings, the pooled TZ and TZS endpoints gave a lower access resistance on average than the WS endpoint, but the difference was not significant. Considering only neurons, the average absolute R_a_ values were more similar across the methods, also with no significant differences. Overall, the relative access resistance R_a_/R_in_ was significantly lower for neurons compared to glial cells (0.78±0.56, N = 136, and 1.5±2.4, N = 44, respectively, p<0.005). Across methods, for neurons there was no significant difference in R_a_/R_in_. For glial cell recordings, R_a_/R_in_ was lower for the pooled TZ and TZS recordings compared to the WS method, but the differences were not significant. In addition to the average R_a_ and the recording duration, the quality of a recording can be assessed by the stability of R_a_. For neurons and glia, ΔR_a_/Δt was larger for the pooled TZ and TZS endpoints as compared with the WS endpoint, but this difference was not statistically significant.

#### Correlation between recording and cellular parameters and access resistance

**Figure 9:**
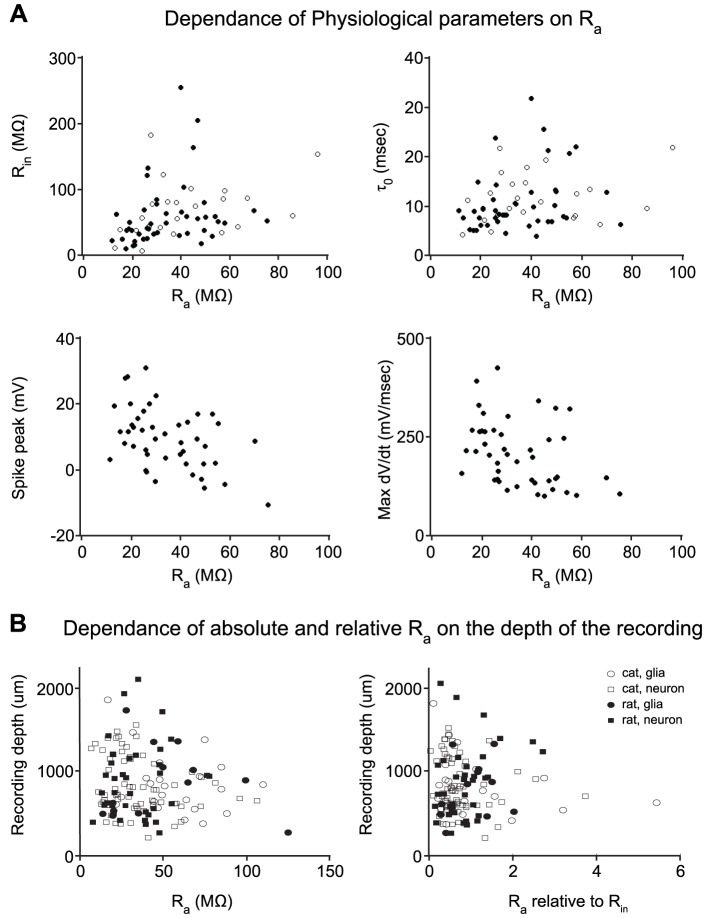
Neuron parameter dependencies on estimated access resistance R_a_. (A) Rat neurons closed circles, cat neurons open circles; Top left, cell input resistance versus R_a_ (r^2^ = 0.08). Top right, cell membrane time constant versus R_a_ (r^2^ = 0.06). Bottom left, peak spike voltage for regular-spiking neurons versus R_a_ (r^2^ = 0.22). Bottom right, peak spike depolarization for regular-spiking neurons versus R_a_ (r^2^ = 0.14). (B) Dependence of absolute and relative R_a_ on recording depth. Left, absolute value of R_a_. Right, R_a_ normalized to the cell input resistance R_in_. Pearson correlation coefficients (r^2^) are calculated over the entire dataset in each plot.

We then examined how access resistance was correlated with various experimental parameters (Figure 9). We first examined how absolute and relative R_a_ depended on the recording depth, and we found no obvious relation between these parameters in either the rat or the cat, nor for neurons or glial cells. This finding is in contrast to previous studies, for example Margrie and colleagues [17] reported that across different species, ages and target areas in the brain, deeper recordings (range of approximately 300 to 4600 microns) were correlated with higher R_a_. While in our study the target cells were confined to a single structure, adult cerebral cortex, there was still a large span of recording depths because of the oblique positioning of the manipulator, ranging roughly between 200 and 2000 microns.

We next examined if R_a_ might influence measured properties of rat and cat neurons (Figure 9). There was no clear dependence between R_a_ and R_in_ or between R_a_ and τ0, suggesting that the span of access resistances in this study allowed reliable estimation of the recorded neuron's passive properties. In contrast, the inevitable low-pass filtering of the electrode is seen in the significant negative correlation between R_a_ and both spike peak and the maximum rate of spike depolarization, restricted to rat regular-spiking neurons as above (Spearman correlation coefficient ρ = −0.45 and ρ = −0.43, respectively; p<0.005, 1-sided z test).

## Discussion and Conclusions

We have described Touch and Zap, a revised method for obtaining the whole-cell patch clamp configuration for neurons and glial cells, here applied to *in vivo* blind patch recordings in rat and cat cortex. The nature of this protocol provides three variants for any given recording (Figure 1): All cases start with a “touch”, with positive pressure in the electrode removed immediately on contact with a target cell. Final whole-cell access is then obtained by either a “zap” by current clamp electroporation alone (TZ here), by mouth suction if there is resealing after initial whole-cell access by electroporation (TZS here), or by suction alone when the attempt of spontaneous sealing and electroporation was abandoned (TS here). We have evaluated the Touch and Zap technique by comparing results with recordings using the classical blind patch method that “washes” the cell membrane after contact, and then achieves gigaohm-seal formation and finally whole-cell access with suction (WS here). We have also described a heuristic method for extracting linear cell properties, including access resistance, which should be straightforward to implement on-line for “real-time” R_a_ estimation while in current clamp mode. Overall, we are able to record cortical neurons and putative glial cells with low and stable values of the access resistance that compare well with values reported in the literature (Table 5).

**Table 5:** Reported access resistance values from *in vivo* whole-cell patch recordings of neurons in mouse, rat and cat cortex, compared with values from the present study.

Species	Age	R_a_ (MΩ)	Reference
Mouse	P28-34	15–40 (17)	[20]
	P21-35	55±4 (19)	[51]
	P28-38	20–60 (12)	[52]
	5–12 weeks	30–100 (20)	[53]
	2–8 month	31±17 (21)	[54]
Mouse & Rat	P21-37 (M), P19-44 (R)	63±(99)	[55]
Rat	P21-36	47±3 (47)	[17]
	Adult	119±12 (10)	[17]
	P28-36	10–75 (48)	[56]
	P17-24	83+90 (62)	[57]
	P17-24	60 (16)	[58]
	3 months	25–60 (21)	[59]
	P19-26	20±21 (122)	[60]
	**Adult**	**35±19 (86)**	**This study**
Cat	Adult	50–200 (25)	[10]
	Adult	100–250 (37)	[61]
	Adult	22±15 (109)†	[36]
	Adult	140±44 (12)††	[34]
	Adult	70.5±39.7 (40)	[62]
	**Adult**	**36±19 (83)**	**This study**

Note the general tendency for smaller access resistances from recordings on younger animals.

† : Recordings limited to R_a_<60 MΩ.

†† : R_a_ taken as the mean of the reported values for the “electrode resistance” corrected with the “residual access resistance” (see table 1 in [34]).

### The challenge of bridge compensation

Because many of our protocols involve the quantitative measurement of biophysical properties using voltage clamp and dynamic clamp, and measuring evoked synaptic conductances, we were interested in assessing R_a_ for the new method. Reliable compensation of R_a_ is critical for these protocols because the impedance of the measuring device is often on the same order of magnitude as the cellular mechanisms under study, and thus can easily distort the measurement. For example, incomplete compensation of R_a_ will lead to an underestimation of evoked conductances, confounding the goal of deciphering excitatory and inhibitory synaptic input underlying post-synaptic potentials. Specifically, a large absolute value of R_a_ has the following consequences. First, as the electrode time constant, proportional to R_a_, becomes closer to the cell time constant, it becomes more difficult to estimate (and then compensate) R_a_. Second, a large R_a_ tends to show non-linear properties, compromising the amplifier's linear bridge and capacitance compensation circuits for large currents. Third, the electrode and the compensation circuits are non-negligible in the voltage-based feedback loop of voltage clamp and dynamic clamp protocols. A large electrode time constant will tend to low-pass filter the measured voltage, and the associated compensation from the amplifier necessarily degrades system stability and dynamic range, especially if electrode non-linearities distort the voltage. It is also important to constrain the relative value of R_a_ with respect to the cell input resistance R_in_: An error in R_a_ compensation will give a voltage error that is proportional to the product of the R_a_ error and the injected currents, the latter being roughly inversely proportional to R_in_. In our experience, these factors put a practical limit of about 2 on the relative access resistance for successful dynamic clamp or voltage clamp protocols.

While we found that the heuristic for extracting linear response parameters and R_a_ gave good fits to the neuron responses, we have less confidence on the glial cell values because their faster membrane time constants are closer to the electrode time constant. On the other hand, assuming that electrode properties for glial cell and neuron recordings are similar (e.g. average R_e_ was only slightly different), the fact that we find similar R_a_ values for the two cell types suggests that the linear glial cell measures reported here are reasonable.

### Assessing recording quality and duration

Qualitative comparisons between electrophysiological methods are difficult since each experimentalist has different, often incompatible, goals. For example, recording durations are determined not only by protocol complexity and data variability, but also by the type of protocol (if reported at all, recording durations in a given publication typically consider only recordings with durations sufficient to achieve the experimental protocol).

Since one of our goals in this paper is to describe a global picture of what can be expected in routine experiments using our approach, we report the duration of essentially all our recordings which, while including a variety of experimental protocols, required reasonable access resistance values. Consequently the distribution of recording durations reported here (Figure 8) represent a lower bound of the maximal duration each cell could be kept in whole cell mode with more permissive constraints. If “passive” monitoring of synaptic potentials and spike times, or basic intrinsic responses by current clamp are needed, then R_a_ properties are less important and long recordings may be achieved more easily. In contrast, as noted voltage and dynamic clamp protocols require low and stable R_a_, which necessarily reduces recording times. Moreover, we included all recordings for which at least one repetition of an I/V protocol was done, i.e., as short as ∼30 seconds, which tends to reduce the average duration.

In summary, since recording duration *per se* was not a criteria for inclusion in the dataset, these distributions provide a practical, and conservative, perspective on what we normally encounter under our experimental conditions. Furthermore, the distributions for neurons include sufficiently long durations (≥ tens of minutes) to carry out complete functional studies *in vivo*. We also note that although the glial recordings are generally shorter than for neurons (similar results were reported in Kelly and Van Essen, 1974 [47], comparing recording times with sharp microelectrodes between glial and neuron recordings), stable recordings which last several minutes are sufficient to measure basic functional properties, as demonstrated by the examples in Figures 5–7. More relevant here is that for our purposes, the glial recording durations were essentially limited by the brevity of the protocols as described above (maximum duration typically several minutes), rather than by recording quality *per se*.

### Advantages of the Touch and Zap method

The Touch and Zap approach is promising for reliably obtaining low and stable access resistance recordings in a broad range of preparations *in vivo* and *in vitro*, even when excellent visualization of the cell and electrode is possible with microscopy. Current clamp electroporation exploiting gigaohm-seal formation may be useful for fully automated patch clamp methods in high-throughput culture or isolated cell protocols, or even *in-vivo* [16]. This method has proved amenable to *in vivo* blind patching in mouse spinal motoneurons (M. Manuel, A. Schramm and D. Zytnicki, personal communication), and two-photon targeted patching of neurons and glial cells in mouse visual cortex (A. Schramm and P. Kara, personal communication).We note that a necessary condition for successful whole-cell patch recording in vivo is a stable preparation with minimized pulsations and healthy cortex, especially with no edema. Again, we refer the reader to the methodological treatments of [17]–[19] for details.

### In vivo patch recordings of putative glial cells

We are interested in the properties of cortical glial cells *in vivo* as well as neurons, to compare intrinsic properties of glial cells with those reported *in vitro*, and to study their voltage and conductance responses to visual stimuli in primary visual cortex. The difficulty of recording glial cells *in vivo* has been noted previously, with the argument that sharp microelectrodes would be advantageous over patch electrodes because of their smaller size and lack of perfusion from electrode solution [43]. Indeed, to our knowledge no other studies have reported whole cell patch recordings of glial cells *in vivo*. Nevertheless, exploiting the whole-cell patch method is important because of superior electrical properties, and the Touch and Zap method facilitates this goal for glial cells. First, the target population for the Touch and Zap recordings of glial cells is more diverse than for the WS method: The input resistances of the Touch and Zap population span a broader range than the WS glial cells, with a significantly smaller mean L_rel_, implying that the Touch and Zap recordings include smaller cells. Second, the proportion of glial cell recordings that used the touch step was almost twice that for the standard WS method (27% vs. 15%), and approaches the estimated proportion of glial cells in somatosensory cortex of the rat (∼38% in [38]). The increased chance of recording glial cells by the Touch and Zap method is likely a direct consequence of a broader target population, suggesting that delicate cells are sensitive to the “wash” step and mechanical rupture for whole-cell access using the traditional approach. The advantage of electroporation is further supported by the greater stability of R_a_ for the TZ versus the other endpoints, all of which use suction at one stage to enter the cell. Finally, the relative access resistance of glial cell Touch and Zap recordings was lower than WS recordings, suggesting that this approach is better suited for quantitative biophysical measurements of glial cell membrane properties *in vivo* [44], up to now much more common for neurons in the *in vivo* setting.

The fast visual responses of putative glial cells that we report here motivate measurement of functionally evoked synaptic conductances, in precisely the same manner as have been made in neurons *in vivo* (reviewed in [45]). Indeed, voltage responses in cat cortical glial cells recorded with sharp microelectrodes *in vivo* have been observed in response to direct thalamic [46] and visual stimulation [47], in the latter case with the responses tuned to stimulus orientation. Calcium transients of cortical glial cells in the barrel cortex of the mouse [48] and visual cortex of the ferret [49] *in vivo* have also been described, with tuning to sensory input reminiscent of neuronal receptive fields. Notably, these responses have relatively slow dynamics (time constants on the order of one and several seconds for voltage and calcium, respectively). Indeed, the standard terminology for glial cell voltage responses is the “slow depolarizing response”, or SDR, and their time scale is consistent with the proposal that these responses mirror changes in the extracellular potassium concentration [50]. In contrast, our preliminary findings suggest that synaptic-like mechanisms allow a more dynamic functional role of cortical glial cells in sensory processing than has been previously assumed. It is of particular interest to compare our recordings with those of Kelly and Van Essen, 1974 [47]. This paper reported that a majority of non-excitable cells (39 out of 54 recordings) showed visual responses typically of a few millivolts in amplitude. The glial responses were slow, but this may be explained simply by the fact that this study employed slow visual stimuli, e.g. sweeping across the receptive field over a second or so, in comparison with the 4 Hz temporal frequencies that we used here.

#### Reduced impact on the recorded cell and network

We propose two reasons why the Touch and Zap method should, in principle, be less invasive as compared to the traditional WS protocol. First, eliminating the “wash” step can only reduce hydraulic perturbation to the cell and local synaptic circuitry. For rat regular-spiking neurons there was a significantly higher maximum spike depolarization rate for the Touch and Zap versus the WS recordings, which supports the hypothesis that the touch step approach is less invasive to the membrane, in particular with respect to sodium channels.

A second reason arises from the use of current-clamp mode. There are two reasons for manipulating the membrane voltage for obtaining whole-cell patch recordings: Gigaseals are facilitated by hyper-polarizing potentials [17], [33], and electroporation to enter a cell requires an applied voltage of three to four hundred millivolts. These manipulations may be done in voltage-clamp mode, and indeed a survey of the experimental literature reveals that this is the standard approach. For example, whole-cell access may be achieved under voltage clamp mode by overcompensating the electrode capacitance (for example “Buzz” button), resulting in fast oscillations bounded by the amplifier's internal supply voltages. However, these voltages highlight a basic challenge, that of reconciling the need to sufficiently perturb the cell to form gigaseals and break the membrane, versus the desire to make delicate intracellular recordings as non-invasively as possible. A critical problem arises when whole-cell access is achieved: if a large potential for forming a seal or for electroporation is supplied by a voltage-clamp circuit, that same holding potential – corresponding to a large hyper-polarizing current - will then be applied to the inside of the cell. This current could damage the recorded cell, even inducing further electroporation in another part of the membrane. In contrast, obtaining the necessary voltages under current-clamp mode in the closed-loop manner described here minimizes cell damage. Specifically, because of seal formation, and thus increasing resistance, the membrane potentials sufficient for both augmenting further seal formation and eventual electroporation may be achieved by currents on the order of a −1.11 nA. Once whole-cell access is achieved the voltage deflections are much smaller because of the much smaller cell input resistance seen by the electrode. These hyper-polarizing deflections of a few tens of millivolts are normally not injurious, at least for short exposures.

#### Technical advantages

We also find the Touch and Zap protocol easier and more reliable, as whole-cell access is achieved to some extent automatically, and thus depends less on experimentalist skill. The approach is typically faster than the standard approach, with successful access typically determined within a minute or two of the electrode entering cortex, and a matter of seconds after cell contact. Finally, the Touch and Zap protocol is efficient, since even if the TZ or TZS endpoint is not achieved, access may still be possible with essentially the traditional approach (here, TS). This flexibility results in an overall higher “hit-rate” for these challenging protocols.
